# Promoting Earth-Based Radar Astronomical Observations of the Moon

**DOI:** 10.3390/s20071874

**Published:** 2020-03-27

**Authors:** Jing Sun, Jinsong Ping, Yuri Bondarenko, Dmitry Marshalov, Fengchun Shu, Jianfeng Cao, Songtao Han, Lue Chen, Wen Chen

**Affiliations:** 1 National Astronomical Observatory, Chinese Academy of Sciences, Beijing 100101, China; jsping@bao.ac.cn; 2 Institute of Applied Astronomy, Russian Academy of Sciences, St. Petersburg 191187, Russia; bondarenko@iaaras.ru (Y.B.); marshalov@iaaras.ru (D.M.); 3 Shanghai Astronomical Observatory, Chinese Academy of Sciences, Shanghai 200030, China; sfc@shao.ac.cn; 4 Beijing Aerospace Control Center, Beijing 100094, Chinachenlue@xao.ac.cn (L.C.); 5 Yunnan Observatory, Chinese Academy of Sciences, Kunming 650011, China; chenwen@ynao.ac.cn

**Keywords:** radar astronomy, moon, circular polarization ratio, radar albedo

## Abstract

Earth-based radar astronomical observations provide information on surface characteristics, orbits, and rotations for a wide variety of solar system objects. Based on compound radio telescopes, both the Chinese VLBI (Very Long Baseline Interferometry) network (CVN) and the Russian VLBI network (Quasar), in cooperation with the Chinese radar transmitters, we present the current ground radar astronomical observations of the moon. The spectrum of the reflected radio signals was obtained and the Doppler frequency shift in bi-static radar mode was measured. Radar albedo of the observed region and power ratios of the reflected signals with left- and right-hand circular polarizations were determined, allowing us to study the radar reflectivity and near-surface wavelength-scale roughness of the moon. Future developments on radar astronomy are also discussed in the paper.

## 1. Introduction

The radar astronomical method is one of the most effective techniques to determine the physical properties and orbital elements of solar system objects. While the other astronomical technique relies on passive measurements of reflected sunlight or naturally emitted radiation, the defining feature of radar astronomy is human control of the artificially transmitted signal. The size, shape, spin period, and surface properties of solar system bodies can be obtained using radar observations [1,2]. The radar astronomical technique can also be used to protect satellites and monitor potentially dangerous space objects.

Usually, the ground radar astronomical observations employ state-of-the-art ground large radio astronomical antennas, powerful radar transmitters, low-noise receivers, and high-speed data-acquisition computers [3]. Progressive updates of the NASA Goldstone and NSF (National Science Foundation) Arecibo systems, primarily by moving to higher frequencies and more powerful transmitters, have made these two compounds the dominant instruments for current radar studies of the solar system [4,5,6,7,8].

The ground radar astronomical observation can also be carried out by means of the VLBI (Very Long Baseline Interferometry) method. Based on the Low Frequency VLBI Network (LFVN) project, the VLBI radar (VLBR) was developed for the investigation of solar system bodies. LFVN has been arranging VLBR experiments since 1999 with the help of the C-band transmitter of Evpatoria RT-70 and the X-band transmitter of Goldstone RT-70. The VLBR combines the radar sounding of space objects with a powerful transmitter and the receiving of radar echoes by an array of radio telescopes in VLBI mode. The VLBR supplements the traditional radar methods with the interferometric reception of a ground-based transmitter’s echo signal reflected from the object. VLBR allows us to measure the variations of proper rotation in Earth group planets and determine the trajectories of planets and asteroids in the Radio Reference Frame. VLBR has been successfully tested in a large series of international experiments: a study of the short–periodic variation of proper rotation for Earth group planets, determining the orbits of asteroids crossing the Earth’s orbit, measuring the space debris population at geo-stationary and high-elliptic orbits [9,10,11,12,13].

Since 2015, the Institute of Applied Astronomy (IAA) of the Russian Academy of Sciences and the Goldstone Deep Space Communications Complex have regularly conducted intercontinental radar observations of Near Earth Objects (NEOs) using the DSS-14 antenna as a transmitter and the RT-32 radio telescopes as receivers, thus performing a “bi-static” mode of radar observation [14,15,16,17,18,19,20].

There are several fully steerable dishes equipped with powerful X-band transmitters in China, which are used for deep spacecraft communications [21,22]. The close collaboration of radar transmitters with the CVN gives us the opportunity to apply the VLBI technique in order to receive and analyze echo signals. The first successful radar detection of the moon was carried out on July 2, 2017. Since 2017, radar observations have been regularly organized at the National Astronomical Observatory (NAOC) of the Chinese Academy of Sciences in cooperation with the Chinese radar instruments to transmit and the radio telescopes of the CVN to receive the echoes. The primary goal of this is the development and validation of the radar astronomical method to obtain information on trajectory parameters, attitude motion, and shape for known objects. International joint observations between the CVN and the Quasar VLBI network have been organized since 2018, mostly focusing on radar observations of the moon.

## 2. Experiments

A collaboration program with the Chinese radar team was initiated in 2017, and a series of experiments were carried out for radar astronomical research of the moon. The radar transmitter and a complex of radio telescopes spaced by hundreds and thousands of kilometers constitute the basis of the experimental facilities. All antennas are pointed at the ChangE-3 lunar lander (CE3) coordinates at 44.12° N, 19.51° W [23] at the Mare Imbrium lunar region, taking into account the signal round-trip travel time. The Chinese radar antenna at station Kashi (Ks) [21] illuminates the moon with a continuous wave (CW) signal at a frequency of 7209.125 MHz in left-hand circular polarization. The carrier frequency is not changed over time and the transmitter power reaches 200 W. The signal reflected from the moon is received with the 40-m Kunming (Km) and 13.2-m Zelenchukskaya (Zv) radio telescopes of the CVN and the Quasar VLBI network in left- and right-hand circular polarizations simultaneously, i.e., in the same (SC) and opposite (OC) circular polarizations as that of the transmitted wave. The echo signal is quantized with two bits and written in digital form and the distribution of echo power as a function of frequency is measured.

In order to carry out the radar astronomical observations, the signal-to-noise ratio (SNR) and the Doppler shift of the carrier frequency are estimated. The motion information of the moon and corrections to the parameters of the Earth’s rotation are formed from the DE436 numerical theory. The coordinates of the antennas, their diameters D, aperture efficiency η, noise temperature $T_{r}$ of the receiving antenna systems, power $P_{t}$, and frequency $f_{0}$ of the transmitter signal as well as the CE3 lander coordinates are specified as input parameters (see Table 1). The parameters of the transmitting and receiving antenna systems are listed in Table 1.

SNR determines whether a radar echo from a distant target is detectable. The SNR is estimated based on a specified configuration of the transmitting and receiving antenna systems and target to be observed. The signal-to-noise ratio is defined as:

(1) $$SNR=P_{r}/\Delta P_{n},$$

where $P_{r}$ is the received echo signal power, and $\Delta P_{n}$ is the noise power’s standard deviation. The power $P_{r}$ of the received echo signal is calculated using the radar equation:

(2) $$P_{r}=P_{t}G_{t}A_{r}\sigma /{\left(4\pi R^{2}\right)}^{2}$$

where $G_{t}$ is the gain of the transmitting antenna, $A_{r}=\eta \pi {\left(D/2\right)}^{2}$ is the effective aperture of the receiving antenna, σ is the radar cross-section in a specified polarization, and R is the distance from the transmitting antenna to the moon. The gain of the transmitting antenna is determined from effective aperture $A_{t}$ and wavelength λ according to the formula $G_{t}=A_{t}4\pi /\lambda^{2}$. The radar cross-section of an object is the projected area of a perfectly reflecting sphere that would return the same echo power at the receiver as the target, and it can be written as $\sigma =\hat{\sigma }A_{proj}$, where $\hat{\sigma }$ is the radar albedo. The projection of the illuminated area of the moon surface can be calculated as $A_{proj}=\pi {\left(l\alpha \right)}^{2}/4$, where α is corresponding antenna beam width and l is a distance from the antenna to the surface of the moon. $A_{proj}$ is decided by the minimum beam widths of the transmitting and receiving antennas. Therefore, the received echo is reflected from the illuminated area on the moon with a diameter of around 503 km for the Km antenna and 1118 km for the Zv antenna, which is much smaller than the lunar diameter.

The noise power’s standard deviation in the bandwidth *B* of the echo is given by:

(3) $$\Delta P_{n}=kT_{r}B/\sqrt{B\Delta \tau }$$

where Δτ is the integration time of the signal on the receiving end and k is the Boltzmann’s constant.

Different parts of a rotating target have different velocities relative to the radar, so the echo will be dispersed taking into account the Doppler effect. In our case, the bandwidth *B* of the echo, or Doppler broadening, is determined by the illuminated area on the surface of the moon. For an accurate estimation of the Doppler broadening, we took 49 points on the Moon surface evenly distributed around the CE3 lander at a diameter of 503 km and 1118 km from station Km and Zv, respectively. The frequency of the received signal is shifted in proportion to the rate of change of distances from the moon to the transmitting antenna v and to the receiving antenna u by a value equal to

(4) $$f_{D}=f_{0}\left(1+\frac{u}{c}\right)/\left(1-\frac{v}{c}\right)$$

Relative radial velocities u and v were calculated for each reflected point using the DE436 lunar libration model on epoch 26 April 2019, 03:00:00 UT. The variation of lunar topography is not taken into account. Then, using Equation (4) we obtained the minimum $f_{D_{min}}$ and maximum $f_{D_{max}}$ echo-signal frequency from the illuminated area on the moon, amounting to 14,596 Hz and 14,631 Hz for Km, and 6608 Hz and 6698 Hz for Zv, which correspond to the Doppler broadening $B=f_{D_{max}}-f_{D_{min}}$ of the echo of 35 and 90 Hz for Km and Zv, respectively.

Our SNR estimations are about 167,000 and 185,000 for Km and Zv correspondingly on 10 s integration time, assuming *B* is set to be 35 Hz and 90 Hz for Km and Zv respectively and radar albedo ${\hat{\sigma }}_{OC}$ of 0.07 for the moon [3]. Such SNR values are enough to register an echo-signal.

The Doppler shift $f_{D}$ of the echo signal is taken into account when carrying out and processing radar observations. The frequency of received signal $f_{echo}$ is determined by the formula $f_{echo}=f_{0}+f_{D}$, where $f_{0}$ = 7209. 125 MHz is the carrier frequency of the radar at the Ks station and $f_{D}$ is obtained using Equation (4) for the CE3 lander position. It should be noted that u and v in Equation (4) are functions of time, so the $f_{D}$ is not a constant. We estimated that the Doppler shift varies in one hour from −13 to −16 kHz for station Km, and it varies from −4 to −9 kHz for station Zv.

## 3. Initial Results

Joint radar observations of the moon started on 26 April 2019 at 02:30:00 UT and lasted for 60 min. The echo signals reflected from the moon were confidently detected by each antenna and written in digital form, which provided reliable data at all stages of processing. After the radar sessions, the raw echo data from each receiving antenna were transmitted to NAOC and IAA for further data processing.

A spectral analysis of the received signal at each antenna was carried out for further calculation of the frequencies. Such analysis aims to measure the frequency difference between the emitted and the received signals. The difference, namely Doppler frequency shift, is conditional on the radial velocity of the object on the path “transmitter-Moon-receiver”. The digital fast Fourier transform algorithm is used to obtain the power spectrum of an echo signal, and the observed echo frequencies are determined using the peaks of the spectra, taking into account frequencies calculated from the precise ephemeris. We found that the echo frequency deviation was −15 Hz for the Km and −10 Hz for the Zv stations, respectively. This may be due to inaccuracies in the coordinates from the center of the radar beam on the moon’s surface.

**Figure 1:**
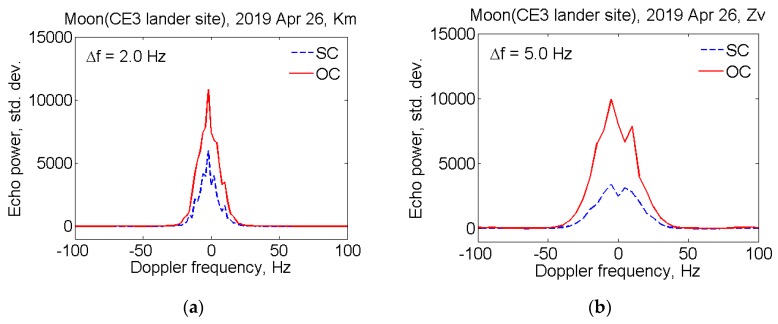
Opposite (OC) (red solid line) and same circular polarizations (SC) (blue dashed line) echo power spectra from the moon obtained at Km (**a**) and Zv (**b**) radio telescopes on 26 April 2019 from 03:00:01 to 03:00:11 UT. The vertical axis shows the amplitude of the received signal in standard deviation of noise level, where zero is the noise expectation value. Horizontal axes display frequency in Hz, with the 0 Hz corresponds to the echo frequency from the ChangE-3 lunar lander (CE3) position. The frequency resolutions are 2.0 Hz and 5.0 Hz for (**a**,**b**) spectra, respectively.

Proceeding from an a priori estimate of the echo-spectrum bandwidth B, the frequency resolution of the spectrum Δf is set, so that the condition Δf≤B is satisfied. The time interval of 0.5 sec and 0.2 sec, i.e., frequency resolutions of 2 Hz and 5 Hz, is also set to obtain the echo spectrum from Km and Zv data accordingly. For each data sample *i*, the Doppler frequency shift $f_{Di}$ of the echo signal is taken into account, so that the echo-signal accumulates at the fixed frequency. The spectra of echo from the Moon, obtained at station Km and Zv, are shown in Figure 1. The red solid and blue dashed lines denote echo power in the OC and SC polarizations, respectively. The echo power is plotted in standard deviations of the noise versus Doppler frequency relative to the estimated frequency of echoes from the CE3 lander position; therefore the zero value corresponds to the noise expectation value.

From the above spectra, it is possible to estimate the real SNR values. Since the echo spectrum is expressed in terms of the standard deviation of noise, the SNR is determined by the sum of SC and OC integrals. Thus, for example, the real SNR value for echo obtained with the Km and Zv are 177,000 and 203,000 respectively, taking into account frequency resolutions Δf of 2.0 Hz and 5.0 Hz.

The profile line width is determined by the rotation of the moon’s reflecting surface, while its change is indicative of the shape asymmetry. The power spectrum bandwidth as a function of time can be used to obtain the spin period in the case of long observation series. As seen from Figure 1, the spectrum widths B for Km and Zv radio telescopes are about 40 Hz and 90 Hz, which coincides with the a priori estimate.

The circular polarization of the signal is reversed after reflection from the plane surface and the maximum power of the reflected signal is expected in the OC polarization, though some of the signal, due to secondary reflections, is received with the same polarization. The circular polarization ratio $\mu_{C}=\sigma_{SC}/\sigma_{OC}$ is a measure of near-surface wavelength-scale roughness [24]. In our case $\mu_{C}=$ 0.44 for Km spectra shown in Figure 1a and $\mu_{C}=$ 0.45 for Zv spectra in Figure 1b, taking into account system noises in the different polarizations.

The radar albedo is one of the most widely used measures of radar reflectivity. The average radar albedo computed for the OC spectra shown in Figure 1 is ${\hat{\sigma }}_{OC}=$ 0.06 for both stations. In calculations we used Formula (1) and data from Table 1, assuming $SNR_{OC}=$ 121,000 and $SNR_{OC}=$ 149,000 on 10 s integration times, B= 40 Hz and B= 90 Hz, $A_{proj}=$ 503 km and $A_{proj}=$ 1118 km for station Km and Zv, respectively.

**Figure 2:**
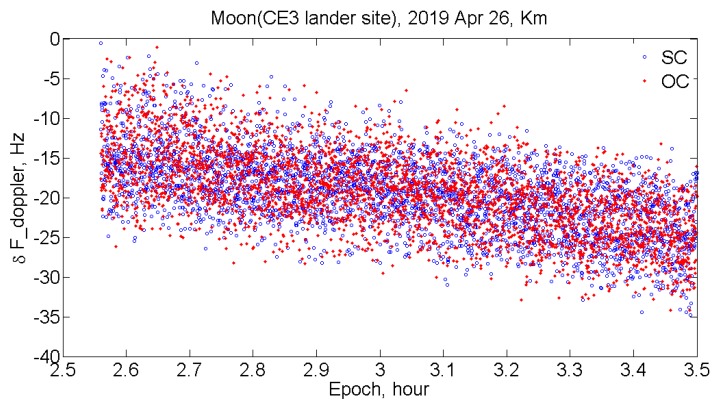
The difference between measured and calculated Doppler frequency shifts for SC and OC echoes, respectively.

As a result of processing the observational data recorded with the Km antenna over the entire session, the power spectra of echo signals from the moon were obtained. The experimental Doppler frequency shifts were determined by the spectrum maximum frequency in bi-static mode. By calculating radial velocity of the moon relative to the transmitting antenna and the receiving antennas, the computed Doppler frequency shift for each of the reception points was obtained. As a whole, the measured Doppler shifts are in good agreement with the calculated values from the lunar ephemeris, and the difference is around 15 Hz. Figure 2 shows the difference between calculated and experimental Doppler frequency shifts over the session, and the standard deviation is approximately 7 Hz.

## 4. Conclusions and Outlook

The signal transmitted at X band by the Chinese radar and reflected from the moon was successfully received with the radio telescopes of the Chinese VLBI Network and the Russian Quasar VLBI network. The obtained results confirm the possibility and effectiveness of the bi-static radar observations of the moon using radio astronomical telescopes as the receiving part of a bi-static configuration. An analysis of the results showed that the SNR and Doppler broadening values of the echo spectra coincided with the a priori estimates. We obtain the circular polarization ratio $\mu_{C}=$0.44 − 0.45 as well as the radar albedo ${\hat{\sigma }}_{OC}=$ 0.06 of the observed Mare Imbrium lunar region, which are consistent with other radar observations [25,26].

It was also shown that the receiving and processing of the continuous wave echo allows us to estimate the value of the Doppler frequency with sufficient accuracy. Following this positive experience, we plan to continue exploring the topic by studying the interference frequency in VLBI mode. The multiplication of signals, recorded in two VLBI-stations, will be carried out to measure the frequency of the interference (fringe rate), which depends on the angular velocity of the investigated object.

If the object is illuminated with a pseudo-noise or the LFM (Linear Frequency Modulated) signal for a given mode, the spatial delay is measured. This delay represents the time difference of the signal propagation from the object to the two receiving telescopes and is determined from the angular coordinates of the object. We also consider the processing of pseudo-noise or the LFM-signal with significantly lower signal-to-noise ratio than in the case of monochromatic signal.

In future, the bi-static radar observations of potentially dangerous asteroids during their approach to the Earth will be carried out. Precise measurements of ephemeris of space debris fragments are important for the prognosis of possible dangerous approaches to operational satellites.

## Figures and Tables

**Table 1:** The Performance Characteristics of the Radio Telescopes.

Antenna	Ks	Km	Zv
Diameter D, m	18	40	13.2
Aperture efficiency η	0.5	0.47	0.7
System temperature $T_{r}$, K	—	96	40
Transmitter frequency $f_{0}$, MHz	7209.125	—	—
Transmitter power $P_{t}$, W	200	—	—
